# Making Implementation Science More Rapid: Use of the RE-AIM Framework for Mid-Course Adaptations Across Five Health Services Research Projects in the Veterans Health Administration

**DOI:** 10.3389/fpubh.2020.00194

**Published:** 2020-05-27

**Authors:** Russell E. Glasgow, Catherine Battaglia, Marina McCreight, Roman Aydiko Ayele, Borsika Adrienn Rabin

**Affiliations:** ^1^Department of Family Medicine, School of Medicine, University of Colorado, Aurora, CO, United States; ^2^Director, Dissemination and Implementation Science Program, The Adult and Child Consortium for Health Outcomes Research and Delivery Science, School of Medicine, University of Colorado, Aurora, CO, United States; ^3^School of Medicine, University of Colorado, Aurora, CO, United States; ^4^Independent researcher, Aurora, CO, United States; ^5^Department of Health System/Management and Policy, Colorado School of Public Health, University of Colorado Denver, Aurora, CO, United States; ^6^Veterans Health Administration (VHA), Washington, DC, United States; ^7^Seattle-Denver Center of Innovation, VA Eastern Colorado Health Care System, Denver, CO, United States; ^8^Department of Family Medicine and Public Health, School of Medicine, University of California, San Diego, San Diego, CA, United States; ^9^Seattle-Denver Center of Innovation, VA Eastern Colorado Health Care System, Denver, CO, United States; ^10^Dissemination and Implementation Science Program, The Adult and Child Consortium for Health Outcomes Research and Delivery Science, School of Medicine, University of Colorado, Aurora, CO, United States

**Keywords:** implementation science, frameworks, rapid, iterative, adaptation, RE-AIM, evaluation

## Abstract

**Introduction:** Implementation science frameworks have helped advance translation of research to practice. They have been widely used for planning and *post-hoc* evaluation, but seldom to inform and guide mid-course adjustments to intervention and implementation strategies.

**Materials and Methods:** This study developed an innovative methodology using the RE-AIM framework and related tools to guide mid-course assessments and adaptations across five diverse health services improvement projects in the Veterans Health Administration (VA). Using a semi-structured guide, project team members were asked to assess the importance of and progress on each RE-AIM dimension (i.e., reach, effectiveness, adoption, implementation, maintenance) at the current phase of their project. Based on these ratings, each team identified one or two RE-AIM dimensions for focused attention. Teams developed proximal goals and implementation strategies to improve progress on their selected dimension(s). A follow-up meeting with each team occurred approximately 6 weeks after the goal setting meeting to evaluate the usefulness of the iterative process. Results were evaluated using both descriptive quantitative analyses and qualitative assessments from interviews and meeting notes.

**Results:** A median of seven team members participated in the two meetings. Qualitative and descriptive data revealed that the process was feasible, understandable and useful to teams in adjusting their interventions and implementation strategies. The RE-AIM dimensions identified as most important were adoption and effectiveness, and the dimension that had the largest gap between importance and rated progress was reach. The dimensions most frequently selected for improvement were reach and adoption. Examples of action plans were summarizing stakeholder interviews for leadership, revising exclusion criteria, and conducting in-service trainings. Follow-up meetings indicated that teams found the process very useful and were able to implement the action plans they set.

**Discussion:** The iterative use of RE-AIM to support adjustments during project implementation proved feasible and useful across diverse projects in the VA setting. Building on this and related examples, future research should replicate these findings and further develop the methodology, as well as explore the optimal frequency and timing for these iterative applications of RE-AIM. More generally, greater focus on more rapid and iterative use of implementation science frameworks is encouraged to facilitate successful translation of research to practice.

## Introduction

It is widely accepted that use of theory improves outcomes, understanding and generalization (1–3) within implementation science as well as other areas. There are many implementation science theories, models, and frameworks that have been used for various purposes (1–4). Our research group has developed, refined, and disseminated the RE-AIM (Reach, Effectiveness, Adoption, Implementation, and Maintenance) framework that has been widely used for evaluation and more recently, planning programs (5, 6). RE-AIM has been found to be useful for both researchers and practitioners (7–9) and for planning as well as end of project evaluations (6). However, with a few exceptions noted below and summarized in the discussion (10–14), to our knowledge, neither RE-AIM nor other implementation science models have been systematically used for, nor specific guidance provided, for mid-course corrections, or rapid assessment and feedback.

If implementation science is to have more impact in real world settings, it needs to become more rapid and iterative (15–17) to address the needs and time frame in which organizations need to make decisions. There have been recent advances in more rapid approaches to qualitative analyses (18–20) and discussion of integrating implementation science with quality improvement procedures to make it more rapid (21–23), but little use of implementation science models to help inform and guide such improvement and adaptations. Many studies track ongoing implementation efforts and report findings (24) using RE-AIM or other implementation science models and outcomes, but few have provided detailed guidance, reported results on or compared stakeholder perspectives on both priorities and progress over time, specific goals set and/or provided tools and resources that can be used by others. As detailed in the discussion, this study extends upon the important efforts above by providing more detail, and reporting application across different interventions, conditions and stages of multiple research projects.

Implementation science models such as Intervention Mapping (25), the Consolidated Framework for Implementation Research (CFIR) (26), RE-AIM (5, 6) and others have been used to plan and guide pre-implementation strategies, but in general, application of these models is not rapid enough to inform during-study adaptation (27). It is also well-documented that context also changes over time (28, 29), that adaptations occur with or without guidance and in ways that are either intervention congruent or not (27, 30), and that sustainment of outcomes almost always requires adaptations (31). Thus, it would help to have a systematic, framework-informed strategy to guide adaptations in response to emerging results and changing context. Such an approach would also be very congruent with and useful for learning health system approaches (32, 33). In summary, we think that rapid learning systems, as well as implementation science research in general, could benefit from systematic and integrated use of frameworks, methods, and iterative processes to evaluate interim progress, ensure that unintended consequences do not occur, and help guide appropriate adaptations.

The goals of this paper are to describe: (1) a team engagement and reflection process to identify RE-AIM dimensions that are most important and most in need of improvement at the current point in the project cycle in each of five Veterans Health Administration (VA) Health System improvement projects; (2) the use of this framework-driven procedure and related data to guide development and execution of an action plan to address key RE-AIM dimensions identified and facilitate mid-course adaptations; and (3) the feasibility and short-term usefulness of this iterative RE-AIM process and directions for future research and practice.

## Materials and Methods

### Setting and Description of the Projects

A detailed description of the project settings and the five interventions has been provided elsewhere (34–38) and is summarized in Table 1. Briefly, four interventions described in this paper emerged from the VA Triple Aim Quality Enhancement Research Initiative (QUERI) (https://www.queri.research.va.gov/) and a fifth VA initiative was funded through the VA Office of Rural Health. The five projects are diverse in the program focus area, clinical problem they address, research and implementation team involved, target population, and the intervention format and delivery. These projects involve different healthcare settings including hospitals, primary care, centralized VA offices, and community settings. The first project, Patient Reported Health Status Assessment, utilizes Interactive Voice Response technology to capture the pre- and post-procedural patient-reported health status for patients receiving elective catheterization laboratory procedures to inform clinical care (35). The second project, Multimodal Pain, addresses barriers and facilitators to multimodal pain care in the VA and designs and implements an intervention based on identified best practices to support primary care providers (38). The third project, Community Transitions, focuses on care coordination of Veterans admitted to non-VA community hospitals for inpatient care, and their transition back to VA primary care in a safe, patient-centered and timely manner (36). The fourth, project, Advanced Care Coordination, aims to improve care coordination for Veterans discharged from community emergency departments by addressing social determinants of health. The fifth project, Rural Transitions, is a proactive, personalized, nurse-led, and Veteran-centered intervention to improve access for rural Veterans to follow-up with their primary care teams following hospitalization at a larger urban VA Medical Center (37).

**Table 1:** Characteristics of five health services research implementation studies.

	**Patient reported health status assessment**	**Multimodal pain**	**Community transitions**	**Advanced care coordination**	**Rural transitions**
Problem addressed	Lack of standardized reporting of patient health status in setting of cardiovascular procedure	Delivering multimodal pain care through tele-mentoring	Transitional care from non-network hospital to network primary care	Transitional care from non-VA community hospital-based emergency department (ED) to VA primary/specialty care	Care coordination for rural Veterans during and post-discharge from a tertiary VHA Medical Center back to their patient aligned care team
Setting	VHA Medical Center	VHA Medical Center, community-based outpatient clinics	VHA Medical Center, community-based outpatient clinics, community hospitals	VHA Medical Center, community-based outpatient clinics, community EDs	VHA Medical Center, community-based outpatient clinics
Intervention	To collect patient-reported health status information before and after percutaneous coronary intervention via an interactive voice response system, and to integrate use of the health status data into routine clinical care	Leveraging data to identify gaps in the use of multimodal pain care, and to train providers in best practices through tele-mentoring	Integrated, non-network hospital discharge care coordination program that includes nurse care coordination and health system changes, including dedicated phone and fax lines for non-network hospitals and Veteran care identification cards	Assess social determinants of health of all Veterans admitted to community ED and discharged home for follow-up care with VA primary/specialty care	A transitions nurse at the VHA Medical Center who prepares patient for discharge and obtains a follow-up appointment, communicates with the patient aligned care team site about the discharge care coordination, follows up with the patient within 48 h after discharge, and engages with the rural primary care provider and registered nurse to ensure continuity of care and information exchange
Implementation strategies	Audit and feedback; facilitation	Audit and feedback; facilitation	Audit and feedback; facilitation	Audit and feedback; facilitation	Audit and feedback; internal and external facilitation; modified rapid Process improvement workshop

At the planning stage of each grant proposal and study, each team had specified key outcomes for the various RE-AIM dimensions. These were slightly modified by the primary investigators at baseline from the measures in their original QUERI proposal. Table 2 provides a summary of the initially established RE-AIM measures by dimension for each project. Other members of the implementation team were not involved in this specification, and several had not yet been hired or assigned to the project at baseline.

**Table 2:** Operationalization of RE-AIM measures by projects.

	**Patient-reported health status assessment**	**Multimodal pain**	**Community transitions**	**Advanced care coordination**	**Rural transitions**
Reach	Number, proportion and representativeness of Veterans: • called by automated calls pre-procedure • who answered the automated calls pre-procedure • reached by automated calls 1 month post-procedure • who answered the automated calls 1 month post-procedure • reached by automated calls 6 months post-procedure • who answered the automated calls 6 months post-procedure Number, proportion and representativeness of cath labs who informed their Veterans of this program	Number, proportion, and representativeness of Veterans with chronic pain care who are seen by providers after providers receive the pain SCAN ECHO training	Number, proportion and representative-ness of Veterans reached by the CHTP program	Number, proportion, and representative-ness of Veterans reached by the ACC program	Number, proportion and representative-ness of Veterans enrolled in TNP Enrollment numbers Rurality GIS maps
Effectiveness	Number, proportion and representativeness of Veterans whose health status is captured and shared to their PCP/Cardiologist pre-procedure: • 1-month post-procedure • 6-months post-procedure • Number, proportion, and representative-ness of providers who utilize reported PROST • outcomes for treatment decision (follow through) • Number, proportion, and representative-ness of Veteran and provider satisfaction using PROST • Number, proportion, and representative-ness of Cath labs satisfaction using PROST	Number, proportion, and representativeness of provider satisfaction with the training (assessed qualitatively) Perception of skills assessment, confidence, perceived knowledge, provider attitude, behaviors Unintended/negative consequences, generalization effects (both positive and negative, at various levels) Assess care utilization using claims data 2 levels: intervention effectiveness, implementation strategy effectiveness	Number, proportion, and representativeness of Veterans: ER utilization after community hospital discharge [among those Veterans who interacted with our program] 30-days re-hospitalizations post community hospital discharge [among those Veterans who interacted with our program] Veteran satisfaction using IVR Veterans who had VA PCP assignment after d/c from community hospitals if no current PCP Veterans who reached out to us post re-hospitalization discharge [Veterans who received our letters]	ER utilization rate after ACC program interaction Veterans 30-day re-admission rate post ACC program interaction [among those Veterans who interacted with our program] Veteran and provider satisfaction with ACC (using IVR) Number, proportion, and representativeness of Veterans who utilized extra visits, services, consults or orders because of ACC involvement	30, 60, 90-days ED Visit Rate, 30-day hospital re-admission rate, death after 30, 60, 90 days 14-days PCP follow up Provider satisfaction Veteran satisfaction Voices of Veterans and providers Relational coordination
Adoption	Number, proportion, and representativeness of Cath labs who follow through suggested program implementation Level of engagement with the program Ability for the Cath labs to identify patients pre-procedure, and identifying ways to reach patients	Organizational factors associated with variation in adoption at various levels Number, proportion and representativeness of providers who received/completed the pain SCAN ECHO training Can you get the right people to participate? Why or why not? Ex.: Understand why we didn't get a high provider reach and what we did about that	Number, proportion and representativeness of community hospitals who inform us of Veteran admission—count this as adoption	Number of times community hospitals notify the ACC program of Veteran ED admission/discharge (specific method important: case manager, fax, phone call) Number and roles of VA providers ACC collaborates with, including any potential referrals	% referrals to CBOCs teams affiliated with TNP Provider satisfaction surveys Provider satisfaction interviews Adaptation interviews with TNs and champions
Implementation	Implementation of core components of the intervention: number of times all or part of the core components are met for each patient Data capture Patient engagement and asking them to call Barriers and facilitators to implementation Adaptations and fidelity tracking Return on investment/cost	Number of SCAN ECHO sessions attended by providers Barriers and facilitators of implementation, contextual factors guided by PRISM Documenting implementation strategies delivery (ex., when and How A&F was delivered, how facilitation was delivered, etc. Economic evaluation Core components, intervention fidelity Adaptations tracking	Number, proportion and representativeness of times community hospitals notify the program of Veteran admission/discharge (specific method important: case manager fax, phone call) Implementation of core components: number of times all or part of the core components are met for each patient Number of medical records received and discharge summaries uploaded Number of follow-up appointments made Number of patients who had the full intervention completed Adaptations made Barriers and facilitators to implementation Cost of intervention Fidelity to the program intervention	Barriers and facilitators to implementation Return on investment/cost Tracking adaptions and fidelity to the program delivery	Theoretical Domain Framework (TNs and champions) Adaptations Tracking using modified Stirman Framework End of program assessment by Cohort 1 site champions Adaptation interviews with TNs and champions Mid-course process assessment Implementation costs; comparison of Cohorts 1 and 2 Final program interviews with Cohort 1 sites
Maintenance	Planned maintenance, including expansion to other sites Unplanned maintenance where VA internalizes program Develop toolkit that other programs can use to engage and implement PROST	Extent to which sites continue to have other providers participate in the SCAN ECHO program after completion of evaluation period Expansion of SCAN ECHO program to other VA sites	Rapid prototyping Local adaptability Intent to sustain	Local adaptability Rapid prototyping Program continuation after funding period ends	Return on investment analysis Program continuation after funding period ends Maintenance Interviews Exit Interviews (if needed)

### Participants and Project Team Members

All implementation study team members from each project were included in the iterative RE-AIM process. We invited a diverse set of participants including the principal investigator, co-investigators, project coordinator, nurses, social workers, research analysts, and research assistants, who were all closely involved with the development, implementation, and evaluation of the interventions. An important aspect of this iterative RE-AIM process is that it gathered diverse perspectives on importance, progress, priorities, and goals. This helped the project team obtain greater team engagement and buy-in when implementing goals emerging from the iterative RE-AIM process.

All meetings were facilitated by one or two members of our QUERI Triple Aim implementation core (RG, CB, MM, BR). The structure of our Triple Aim QUERI Center is such that an Implementation Core team co-led by Drs. Glasgow and Rabin and coordinated by Ms. McCreight, functions as an overarching methodological and support unit advising all projects. Ms. McCreight also serves as liaison between the Implementation Core and individual projects, as she also plays roles on each project team.

### Overview of Iterative RE-AIM Process

The iterative RE-AIM process was conducted separately for each project and involved four steps. Step 1 involved use of a regularly scheduled team meeting during which (a) the implementation science team members explained the purpose of and steps involved in the iterative RE-AIM process, and (b) the project team reviewed the initial operationalization of RE-AIM dimensions developed at the beginning of the project, and then (c) discussed the status of their project on the various RE-AIM dimensions. Step 2 took place at the conclusion of this meeting, in which team members were then asked separately and confidentially to provide ratings on each RE-AIM dimension in terms of (a) its importance at the present stage of the project and (b) their perception of progress to date on that dimension. Step 3 involved a second team meeting, also facilitated by members of the implementation science team, during which the team reviewed the ratings summarized from the individual rating sheets. A group engagement, reflection and discussion process was used to identify one to two key RE-AIM dimensions on which to focus and set specific, measurable, attainable, relevant and timely (SMART) goals (39), and action plans for these dimension(s). Finally, Step 4 involved a follow-up interview with the PI and project manager for each project regarding their progress on the implementation of the SMART goals, and collect data on the feasibility and usefulness of the iterative RE-AIM process.

### Step 1: Team Meeting #1: Preparation and Initial Discussion

Each project team spent one of their regularly scheduled team meetings for this step. These meetings lasted approximately 1 h, involved all project team members and were facilitated by one or two members of the Implementation Core. The main activities for this meeting were:

1. Introduction/general overview and 5-min description of the purpose of the meeting and the iterative RE-AIM process.
2. Review of the pragmatic definition of each of the RE-AIM dimensions and how they had been operationalized for this project (Table 2).
3. General discussion of the status of the project as it related to the RE-AIM dimensions; and an explanation and distribution of a rating sheet to each team member asking about the importance of and progress on each RE-AIM dimension at the current point of their project. While PIs were familiar with these pragmatic RE-AIM definitions and operationalization plans, other members of the team were less or not at all familiar; and benefited from a discussion of these concepts.

### Step 2: Ratings on the Importance of and Progress With the Different RE-AIM Dimensions

As a follow up to the first team meeting, team members were asked to fill out the above rating sheet (Appendix 1) independently between meetings. Two main questions were asked on the rating sheet: (a) how important is each dimension to this project at this time? and (b) how is the project doing on each dimension to date? Team members were asked to use a five-point Likert scale (1 = not important (or not satisfied); 2 = somewhat important or satisfied; 3 = important (or satisfied); 4 = moderately and 5 = extremely important or extremely satisfied). Participants were also encouraged to add comments or examples that supported their rating. For ratings of progress, teams were instructed to use both any objective data available (e.g., participation rates to that point for reach; fidelity checklist data for implementation), and their subjective impressions concerning improvement to date compared to the initially established project goals. Team members were asked to rate RE-AIM dimensions independently and confidentially to allow for unbiased, equal input from each member of the team.

**Figure 1:**
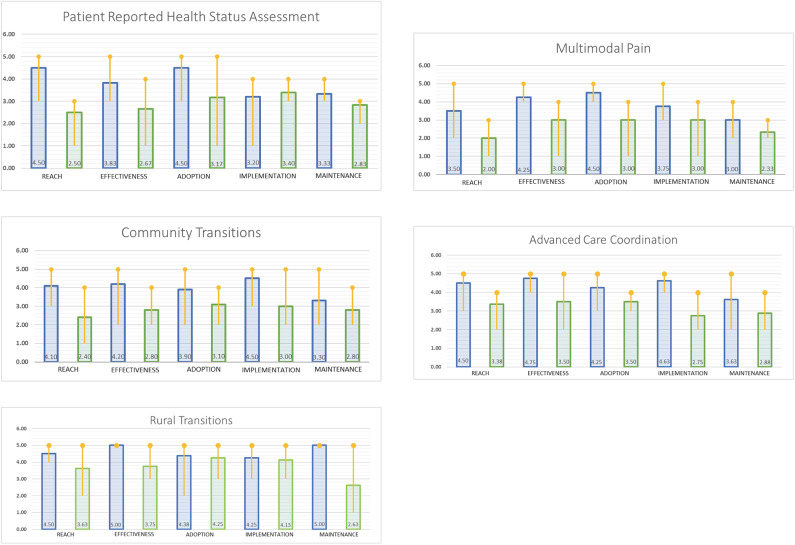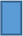 Indicates the rating of the current importance of the RE-AIM dimensions. 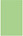 Indicates the rating of satisfaction with progress on the RE-AIM dimensions.

Results from the surveys were analyzed between steps 2 and 3. These results were summarized for the team using simple statistics and visually displayed using histograms at the second team meeting. These histograms displayed the team's cumulative ratings in three different ways including median ratings and variability across raters (Figure 1) on (a) importance, (b) progress on each RE-AIM dimension, and (c) the gap comparing importance and progress ratings on each dimension (three figures per project). All de-identified comments made on the rating forms were added verbatim to the summary report and, presented to each team before meeting #2.

### Step 3: Team Meeting #2: Review Ratings and Goal Setting/Action Planning

A second team meeting focused on review of the summary reports generated from the individual ratings; and goal setting/action planning based on these. During this one-hour session, the following activities were conducted:

1. Reiteration of the purpose of the iterative RE-AIM process and that day's meeting.
2. Distribution and facilitated team discussion of the summary visual displays of rating data and the open-ended comments. Each team member received a copy of both their own ratings and the team summary. The group sequentially reviewed and discussed each of the three displays of their results.
3. Team discussion of and decision on which RE-AIM dimensions should be identified for improvement at that stage of the project based on the information provided. Project teams were asked to agree on one to two RE-AIM dimensions to address at that project stage. We made an a priori decision to limit the focus at a given time point to one or two RE-AIM dimensions given limited resources and the multiple ongoing responsibilities and competing demands of various staff.
4. Goal setting and action planning for the selected RE-AIM dimension(s). Team members were asked to brainstorm possible strategies and specific activities they could use to improve their success on the relevant RE-AIM dimension(s). Then they were asked to create SMART goals and action plans. A template for SMART goal-setting (Appendix 2), and a list of sample action strategies to enhance each RE-AIM dimension were provided to the team. These plans specified which team members were going to do what actions by what date.

Field notes from team meetings were collected to document discussions as well as to record feedback and observations related to the iterative RE-AIM process. After the second meeting, one implementation core member (MM) completed any unfinished items based on the team discussion, and returned the team goal setting/action plan document to all team members within one week after the second team meeting.

### Step 4: Follow-Up on RE-AIM Goals and Evaluation of the Process

For each team, a follow-up session was conducted with the PI and project coordinator approximately 6 weeks after the second meeting. During this 30-min debriefing meeting, data were collected about the team's progress on their SMART goals and intention to revise or continue work on these goals. We also collected ratings of and comments on the usefulness and level of implementation of the iterative RE-AIM process as well as recommendations for improvement (1—not at all; 3—somewhat; 5—extremely useful/completely implemented).

### Data Analyses

Results were evaluated using both descriptive quantitative analyses and qualitative assessments from narrative data and meeting notes. We used matrix analysis (40) to describe and summarize narrative data from surveys and field notes to identify salient themes on each step of the iterative RE-AIM process and creation of the SMART goals and action plans. Matrix analysis is used to summarize qualitative data in a table of rows and columns, for comparison of coded data in cells and observe themes as they emerge. Data from the rating surveys (Step 2) were summarized using simple descriptive statistics (e.g., means and medians) and visual displays. This study was not considered research according to VA Office of Research Oversight policy 1058.05, therefore ethical review and approval was not required in accordance with the local legislation and institutional guidelines.

## Results

Table 3 provides a summary for each project of the current point of time in the project cycle, the number of team members participating, and the roles of participants in the team meetings. The results of the iterative RE-AIM assessment are described for each step of the process as outlined above. During Step 1 (meeting #1) there was a median of seven team members with diverse roles who participated in two team discussions (range= 4–10). Our observations indicated that there was active participation and general equity of discussion across team members. The process and RE-AIM dimensions were deemed understandable for team members, including those who were not directly involved in evaluation or specification of the initial RE-AIM measures.

**Table 3:** Information on participants by project.

	**Patient-reported health status assessment**	**Multimodal pain**	**Community transitions**	**Advanced care coordination**	**Rural transitions**
Current point of time in the project cycle at the time of the assessment	Implementation/Expansion	Pre-implementation	Implementation/Expansion	Implementation/Expansion	Maintenance
Number of participants who completed the assessment (Step 2)	6	4	10	8	8
Number of participants in the discussion (Step 3)	7	4	9	6	9
Role descriptions	PI, project manager, quantitative lead, database programmer, qualitative lead, qualitative analysts	PI, PM, qualitative lead, RA	PI, PM, TN, SW, qualitative lead, qualitative analysts, health economist, clinical consultant	PI, PM, SW, qualitative analysts	PI, PM, RA, qualitative analysts, quantitative lead, quantitative analysts, database programmer

*PI, principle investigator; PM, project manager; RA, research assistant; SW, social worker; TN, transitions nurse*.

There was variability in the RE-AIM dimensions identified as most important and on progress ratings across the different projects. Table 4 summarizes ratings and identifies the most important dimension(s) and rated progress on each dimension by project team. There was a range of RE-AIM dimensions considered most important (Effectiveness, Reach, or Adoption). The Maintenance dimension was generally rated as less important, likely because most projects had not reached the maintenance phase of their project's life cycle.

**Table 4:** Average ratings of importance and progress by project.

**Project**		**Patient-reported health status assessment**	**Multimodal pain**	**Community transitions**	**Advanced care coordination**	**Rural transitions**	**Average rating across all projects**
**RE-AIM dimension**							
Reach	Average rating of importance	4.50	3.50	4.10	4.50	4.50	4.22
	Average rating of satisfaction with progress	2.50	2.00	2.40	3.38	3.63	2.78
Effectiveness	Average rating of importance	3.83	4.25	4.20	4.75	5.00	4.41
	Average rating of satisfaction with progress	2.67	3.00	2.80	3.50	3.75	3.14
Adoption	Average rating of importance	4.50	4.50	3.90	4.25	4.38	4.31
	Average rating of satisfaction with progress	3.17	3.00	3.10	3.50	4.25	3.40
Implementation	Average rating of importance	3.20	3.75	4.50	4.63	4.25	4.07
	Average rating of satisfaction with progress	3.40	3.00	3.00	2.75	4.13	3.26
Maintenance	Average rating of importance	3.33	3.00	3.30	3.63	5.00	3.65
	Average rating of satisfaction with progress	2.83	2.33	2.80	2.88	2.63	2.69

In terms of satisfaction with progress, teams generally rated Adoption and Implementation dimensions highest, with Reach usually receiving the lowest ratings. Combining these data resulted in a visual display of the “gap” between importance and progress, which was consistently the largest for the Reach dimension. Figure 1 illustrates the team members' average score for importance and progress by project as well as the gap between importance and progress ratings.

### Qualitative Results

Examples of participant comments written on the survey to support the ratings included:

- **REACH**: *Continue outreaching current hospitals and enrolling new ones when appropriate. Work on education with community providers on inclusion and exclusion criteria (Advanced Care Coordination project)*.
  *At this point, providers have just started to participate. Reach to Veterans is important, but we can't reach Veterans without reaching the providers first (Multimodal Pain project)*.
- **EFFECTIVENESS**: *It has been hard to measure effectiveness without reaching adequate amount of reach (Community Transitions project)*.
- **ADOPTION**: *The success of the implementation also depends on the engagement and participation of the catheterization laboratory teams (Patient-Reported Health Status Assessment project)*.
- **IMPLEMENTATION**: <*Rural Transitions*> *is making efforts to track and measure our implementation efforts and how effective each is (Rural Transitions project)*.
- **MAINTENANCE**: *Much of maintenance is out of our hands*-<*Rural Transitions*> *has made many efforts to assist each site with maintenance; cost benefit analysis may strengthen this dimension (Rural Transitions project)*.

### Team Goals and Action Plans

Although there was variability, most teams selected Reach as one of the dimensions to target (Table 5). Three teams selected two RE-AIM dimensions to target and the other two focused solely on Reach. Teams most often chose reach and adoption dimensions as needing improvement. Table 5 summarizes SMART goals and action plans developed for each RE-AM dimension the team selected. Examples of reach action plans were “re-engaging key stakeholders to solicit their ideas to reach more participants” and “revising participant exclusion criteria.” An example of an adoption action plan was to conduct chart reviews to closely track adoption.

**Table 5:** RE-AIM Dimension(s) chosen for improvement and key phrases from project action plans by project.

**Project name**	**RE-AIM dimension to focus on**	**SMART goals and action plans**
Patient-reported health status assessment	Reach adoption	1. Conduct workflow assessments to learn where it would fit and how 2. Perform chart review to learn about actions taken after decline status note in the EMR
Multimodal pain	Effectiveness adoption	1. Effectiveness: summarize feedback from semi-structured interviews with providers and review for opportunities to improve program sessions; share the feedback with operational partners 2. Adoption: inform providers of the upcoming sessions 3. Engage/re-engage with program stakeholders for assistance and guidance
Community transitions	Reach	1. Conduct in-services with community hospital to educate about the program enrollment criteria 2. Interview other investigators about how they approach REACH in their projects 3. Consider giving out Veterans program cards pro-actively 4. Review and revise program exclusion criteria
Advanced care coordination	Reach	1. Schedule and conduct educational in-services in participating community hospitals 2. Program social worker to identify best practices of approach at each participating community hospital
Rural transitions	Reach maintenance	1. Review existing literature and plan to collect and analyze real-time return on investment-type data 2. Access operational data and performance measures to compare with program outcomes 3. Discuss with site champions about what leadership and stakeholders need to sustain the program

Field notes from meeting #2 revealed that team members were not surprised by the summary ratings of importance and satisfaction with progress on different RE-AIM dimensions, as they were consistent with their impressions of program challenges and priorities at that time. For example, the Rural Transitions project was beginning the dissemination phase and was largely focused on maintenance efforts; while Multimodal Pain and Patient-Reported Health Status Assessment teams were largely concerned with adoption prior to the assessment process. Additionally, team members discussed how potential improvements in one dimension (e.g., Reach) could lead to impacts on other dimensions (e.g., Effectiveness).

Follow-up assessment meetings were held on average 6 weeks after Meeting #2 with one meeting taking place 15 weeks after the group session due to PI availability. At the time of the follow up meeting, all teams had (a) completed specific SMART goals/action plans with accountabilities specified; and (b) implemented or attempted to implement this plan. Average ratings of the extent to which the plan was implemented was 3.88 on a 5 point scale (1= not at all; 3= somewhat; 5= completely). Teams rated the iterative RE-AIM assessment as being useful (average of 4.25 on the 5 pt. scale of usefulness; 1= not at all; 3= somewhat; 5 = extremely).

The teams were all satisfied with the iterative RE-AIM approach and pragmatic tools. They were implementing action plans based on SMART goals and waiting to evaluate the impact of these on the chosen RE-AIM dimensions. Four out of five project teams commented that it was too early to assess progress on the SMART goals/action plans; the fifth project interviewees reported that they could not move forward due to the exit of their operational partner. Additionally, teams suggested that it would be helpful to conduct the RE-AIM assessments throughout the project phases at regular intervals and suggested a 6-month interval. They felt that this process would help evaluate project progress, address program data collection challenges, and inform adaptations to interventions and implementation strategies. They commented that the focus may shift from one RE-AIM dimension to another over time, resulting in different ratings depending on context and project priorities.

Interviewees also shared lessons learned through the iterative RE-AIM assessment. These included that they were surprised and relieved that they would not need to focus on all the RE-AIM dimensions at once and that it was acceptable to prioritize different dimensions at different phases of the project. For example, Reach was a priority in the implementation/expansion phase and it was reasonable to prioritize Maintenance when the project was further along. Additionally, projects reported experiencing stalls during the implementation phase. The iterative RE-AIM assessment was felt to be useful to overcome barriers and to look for solutions to keep the projects moving forward.

## Discussion

The rapid and iterative RE-AIM assessment and action planning process was feasible and rated as useful for project teams. All five projects found the assessment and planning activities to be understandable and relevant. It is well-established in implementation science that adaptations are going to happen (27, 28, 30) and this approach provides one way to assist in making adaptations purposeful, conceptually based, and data-driven.

The review and reflection process involved was relatively efficient; conducted during two regularly scheduled team meetings and required very little participant work outside of these meetings. The RE-AIM assessment and adaptation process involved all team members and was effective in creating buy in and common goals. There was a balanced discussion and input from team members from a variety of positions and roles, thus supporting and enhancing team science processes (41). The activities were rated as useful and provided teams with a structured and systematic way to assess progress and share perceptions from their different perspectives. This reflection process has recently been reported (23) to be an important aspect of assessment processes that are valued by implementation teams and helpful to inform progress.

There was variability across teams as to which RE-AIM dimensions were most important at that stage in the study, but most felt that Maintenance was less important. While our implementation science team made the decision not intervene to guide discussion or priority setting, these results suggest the opportunity in future applications of this process to point out the importance of designing for sustainability (29, 31), rather than waiting till the end of the project. Most projects reported the least satisfaction with their progress on Reach; their ratings indicated this was the dimension on which there was the largest gap between what they originally planned and what they had achieved; and most teams included Reach as one of the RE-AIM dimensions targeted for mid-course improvement. This focus on Reach is important, both from a health equity perspective (whether the most vulnerable and highest need Veterans were participating), and in terms of population health impact, which cannot be substantial if only a small or unrepresentative portion of the targeted population is reached.

Consensus was achieved among different team members on their perspectives of relative importance and satisfaction with progress on different RE-AIM dimensions. The facilitator-led discussion was informative and useful for team members to hear each other's perspectives. Part of the success and positive ratings may have been because the investigators listened to all team members input and did not dominate the discussion (41). The process might not have been as productive with projects and teams that are more hierarchical. This activity seemed to be a good way to allow for some protected time for team reflection, and to address both progress to date and the longitudinally changing context (1, 29). More generally, the study of adaptions to interventions and implementation strategies during a project is still relatively new and there is not consensus on whether changes to a study protocol should be encouraged or just observed and documented (30).

Adaptations are going to occur whether investigators ignore them or even suppress information on their occurrence (30), thus it makes sense to help to make adaptations fidelity and conceptually consistent rather than haphazard (27). It is still critically important to carefully document and report both fidelity and adaptations for transparency and replication purposes (34, 42), and this mid-course assessment and correction process can help increase reporting on and transparency regarding adaptations.

Prior studies have included some of the elements of our approach in this report. Specifically, Paone (13) used RE-AIM to observe, document, and analyze the implementation experience, as well as the perceived value of and satisfaction with an evidence based program for spousal caregivers in 14 Minnesota organizations. Quarterly reports generated by the consultants provided narrative information on progress and barriers using a mixed-methods assessment of strategies using the five RE-AIM dimensions. In Kwan et al. (12) findings from initial quantitative analysis (e.g., low reach) informed topics for RE-AIM focused interviews and focus groups. In turn, findings from interviews and focus groups informed both practice process improvement and subsequent evaluation priorities. Quinn and colleagues (14) used existing literature and expert consultation to translate and iteratively adapt the RE-AIM framework across several stages of the NIH Clean Cooking Implementation Science case study project while also developing checklists to guide investigators at each stage. Hill and colleagues (11) pilot tested their adapted pediatric weight management intervention iChoose, in 3 iterative phases delivered initially by research partners, then co-delivered by research and community partners, then delivered by community partners. The RE-AIM framework was used to plan and evaluate the iChoose intervention across all waves with assessments at baseline, post program (3 months), and follow-up (6 months). Finally, Forman et al. (10) used the RE-AIM QuEST formative evaluation to identify real-time implementation barriers and explain how implementation context may influence translation to additional settings.

Our iterative RE-AIM assessment and adaptation process is both similar to and different from more frequently used quality improvement (QI) methods (21, 22). Like QI, it is intended to assess progress and guide modifications that can be tested. Although iterative, it is much less rapid than most QI approaches, but it is conceptually based, and explicitly focuses on multiple implementation outcome dimensions important for population health and overall program success (5, 43). A similar, although purely qualitative approach has been suggested by Finley and colleagues in the form of periodic reflections (23).

This study extends related work using RE-AIM for similar purposes by having a more specific, primary and systematic focus on the iterative use of RE-AIM. It adds to the literature by detailing a specific, step by step protocol, using systematic goal-setting, independent ratings by various team members, reflecting on the assessment of both progress and priorities using a standard rating form, evaluating the (short term) impact of the resulting adaptations, and providing scales, guides and resource materials for others interested in this process.

This activity based on implementation science principles and outcomes is also one way to support and operationalize a learning health system (32, 33); and an approach that does not require many resources or much staff time. This is because of the focus on well-defined implementation outcomes and the relative intuitiveness and transparency of the RE-AIM model and measures (8). It is also a way to help teams discuss and focus on “value”- that is, to reflect on whether they are investing resources on and achieving results on what is important (within the confines of RE-AIM implementation outcomes). The observation that the focus might shift during the lifetime of a project is also a critical contribution.

This study has both strengths and limitations. Limitations include the relatively small number of teams and sample size; and that all were projects coordinated from one VA medical center. Also, at least some members of each team were familiar with and had used RE-AIM at the proposal stage. Future directions should include replication in other VAs and non-VA settings and projects that did not use RE-AIM in their initial proposal. This study did not include a control condition and there is clearly a need for more formal and empirical evaluation of the long-term impact of the process. Although the activity explicitly involved all implementation team members, it did not engage Veteran patients or operational leader partners. The iterative RE-AIM process appears helpful in directing mid-course adjustments, but we did not experimentally compare this process to other approaches such as QI or use of other implementation science frameworks. Future research should assess the impact of different timing and intensities of iterative assessments using comparative effectiveness designs and including formal cost analyses (44, 45).

Strengths of this paper include the novel idea of guiding adaptations through rapid and collaborative application of a widely used implementation science framework and the mixed methods assessment. The RE-AIM based evaluation was successfully implemented across five diverse projects, different content areas, at different points in their projects, and with different teams. The pragmatic approach seems to engage team members and appears to be replicable. Finally, our materials are publicly available in the Appendices.

## Conclusions

The use of this RE-AIM based approach was feasible, relatively efficient and seemed to facilitate both engagement of team members having different roles, and mid-course adjustments. Similar rapid assessment and adaptation approaches could be conducted using other implementation science frameworks and comparing different frequencies and intensities of facilitation.

## Data Availability Statement

All datasets generated for this study are included in the article/supplementary material.

## Ethics Statement

This study was not considered human subject research according to VA Office of Regulatory Oversight policy 1058.05 and was designated as quality improvement by the VA Office of Rural Health, therefore ethical review and approval was not required in accordance with the local legislation and institutional guidelines. This is because subjects were not individually randomized, no identifying data were collected from participants and the interventions were done system wide as part of regular care. Therefore, written informed consent for this study was not required in accordance with national legislation and institutional requirements.

## Author Contributions

RG and BR initially conceptualized the study. MM organized the database and performed the statistical analysis. RG wrote the first draft of the manuscript. All authors contributed to conception and design of the study, drafted sections of the text or tables and figures, contributed to manuscript revision, read, and approved the submitted version.

## Conflict of Interest

The authors declare that the research was conducted in the absence of any commercial or financial relationships that could be construed as a potential conflict of interest.
